# Glioma Stem Cells—Features for New Therapy Design

**DOI:** 10.3390/cancers16081557

**Published:** 2024-04-19

**Authors:** Nives Pećina-Šlaus, Reno Hrašćan

**Affiliations:** 1 Laboratory of Neuro-Oncology, Croatian Institute for Brain Research, School of Medicine, University of Zagreb, Šalata 12, 10000 Zagreb, Croatia; 2 Department of Biology, School of Medicine, University of Zagreb, Šalata 3, 10000 Zagreb, Croatia; 3 Department of Biochemical Engineering, Faculty of Food Technology and Biotechnology, University of Zagreb, 10000 Zagreb, Croatia; reno.hrascan@pbf.unizg.hr

**Keywords:** brain cancer, cancer stem cells, genetics, glioma, tumor plasticity, invasion, resistance to therapy, tumor microenvironment, Wnt signaling, glioma heterogeneity

## Abstract

**Simple Summary:**

Gliomas are deleterious central nervous system tumors that harbor cellular heterogeneity and infiltrative capabilities. They are biologically aggressive and highly invasive tumors that lack efficient treatment. Glioma stem cells (GSCs) are a subpopulation of cancer stem cells (CSCs) with the ability for self-renewal that is responsible for tumor plasticity. They show tumor-initiating properties, are influenced by genetic drivers and display great migratory abilities. GSCs engage in a synergistic relationship with the surrounding tumor microenvironment to promote tumor progression and therapy resistance. A great effort is under way in order to find ways to eliminate or neutralize GSCs.

**Abstract:**

On a molecular level, glioma is very diverse and presents a whole spectrum of specific genetic and epigenetic alterations. The tumors are unfortunately resistant to available therapies and the survival rate is low. The explanation of significant intra- and inter-tumor heterogeneity and the infiltrative capability of gliomas, as well as its resistance to therapy, recurrence and aggressive behavior, lies in a small subset of tumor-initiating cells that behave like stem cells and are known as glioma cancer stem cells (GCSCs). They are responsible for tumor plasticity and are influenced by genetic drivers. Additionally, GCSCs also display greater migratory abilities. A great effort is under way in order to find ways to eliminate or neutralize GCSCs. Many different treatment strategies are currently being explored, including modulation of the tumor microenvironment, posttranscriptional regulation, epigenetic modulation and immunotherapy.

## 1. Introduction

Gliomas are deleterious central nervous system tumors that harbor cellular heterogeneity and infiltrative capability. They are biologically aggressive and highly invasive tumors that lack efficient treatment. The complex dynamics of their progression enables them to escape from surgical resection, which is the primary approach in multimodal treatment, together with radiotherapy and chemotherapy. For these most frequent primary brain tumors, the prognosis and survival rate is poor [1], with the five-year survival rate being less than 10%. The incidence of glioma is approximately 6 per 100,000 people worldwide. Age, sex and ethnicity influence the incidence [2], and the mean age of diagnosis is 65. Although the search for the originating cell in glioma etiology is still ongoing, studies report that astrocytes, ependymal cells and oligodendrocytes may potentially be the initiating cells in glioma.

Gliomas can be classified into different malignancy grades based on molecular characteristics, histology and prognosis. Thus, the World Health Organization (WHO) [3,4] divides them into low-grade gliomas (grades 1 and 2), with low proliferative capacity, and high-grade gliomas (grades 3 and 4), with a high proliferative rate, poor prognosis and aggressive phenotype [5]. The most malignant and deadliest glioma is glioblastoma multiforme (GBM), a grade 4 glioma [6,7]. Overall, gliomas account for almost 81% of primary brain malignancies [8,9]. Malignant gliomas are the third most prevalent cause of cancer-related deaths in persons aged 15–34 years [10]. According to the recent WHO CNS5 classification update, diffuse astrocytic gliomas have now been subdivided according to their molecular signatures [11]. An important criterion for adult-type diffuse glioma classification is the mutational status of the genes *IDH1* and *IDH2* that code for key Krebs cycle enzymes isocitrate dehydrogenase [12]. The most frequent of these mutations (accounting for more than 80%) is the missense mutation *IDH1^R132H^*. This results in the substitution of arginine to histidine at residue 132. Thus, gliomas are now divided into *IDH* mutant or *IDH* wild-type. *IDH* mutant gliomas are associated with younger age, as well as a much better disease outcome [13], while *IDH* wild-type carries a poorer prognosis. Along with help in diagnostics, this is also important for the treatment choices and follow-up [14]. 

Relying on genetic findings, the updated classification by WHO CNS5 highlights that glioblastomas comprise only IDHwt tumors. Accordingly, diffuse gliomas can be distinguished as IDHm astrocytoma, IDHm and 1p/19q-codeleted oligodendroglioma, and IDH1wt glioblastoma. In addition, IDHwt diffuse astrocytomas in adults are classified based on molecular parameters as glioblastomas, even when histological high-grade features of glioblastoma are absent. These parameters include one or more of the following three genetic features: (1) the gain of entire chromosome 7 combined with loss of entire chromosome 10, (2) *TERT* promoter mutations and (3) *EGFR* amplification [15]. 

On average, at the time of diagnosis, patients with primary glioblastoma are 64 years old [16], while secondary glioblastomas are diagnosed in adults aged 45 years or younger [17]. As glioblastoma incidence increases with age [18] the number of patients is expected to rise with the growing elderly population. 

Molecularly, glioblastoma is very diverse and presents a whole spectrum of specific genetic and epigenetic alterations, which was initially illustrated in the first comprehensive analysis published by The Cancer Genome Atlas (TCGA) project (Cancer Genome Atlas Research Network, 2008) [19]. At the genetic level, they harbor whole or partial chromosome gains or losses, patterns of somatic mutations, amplification and deletion, while epigenetic mechanisms present methylation or demethylation and transcriptional interference [20]. Later, as a result of molecular profiling, four glioblastoma subtypes were defined—classical, proneural, neural and mesenchymal—each with its own specific molecular properties [21,22]. Clinical presentation of the disease is usually short, ranges from three to six months before diagnosis [10] and with unspecific symptoms. Standard treatment is a combination of surgical tumor removal, followed by radiation and chemotherapy with the alkylating drug temozolomide. The tumors are unfortunately resistant to available therapies and the survival rate is poor [23]. The cellular heterogeneity and infiltrative capability of glioblastoma make complete surgical resection almost impossible. In 75–90% of the cases, glioblastoma typically recurs within one year despite the aggressive treatments (and within 2–3 cm of the margin of the original lesion [10]. The five-year life expectancy of glioblastoma patients is 9.8% in the USA [24] and 2.7% in Europe [25], while ten-year survival is observed in less than 1% of the cases [26]. To improve patient outcomes, numerous alternative approaches such as tumor treating fields [27,28], gamma knife radiosurgery [29] and immunotherapy [30,31,32] are constantly being explored by the research community. Despite these advances, understanding, preventing and treating glioblastoma is one of the most important challenges of neuro-oncology and remains a public health issue.

The great heterogeneity of gliomas, both genetic and morphological, is still not fully understood. It has been shown that diverse cell populations can be found within the single tumor causing intra-tumoral heterogeneity. One of the unanswered questions is also the progression of higher malignancy grades from tumors of lower grades. However, one of the explanations for the glioma significant intra- and inter-tumor heterogeneity, as well as its resistance to therapy, recurrence and aggressive behavior, lies in the small subset of tumor-initiating cells that behave like stem cells, known as cancer stem cells (CSCs). Several studies have identified glioma cancer stem cells (GCSCs) that are similar to other types of cancer cells [33,34,35]. The specific features and biological behavior of GCSCs will be highlighted in the following chapter. 

## 2. Features of Glioma Stem Cells

**Figure 1:**
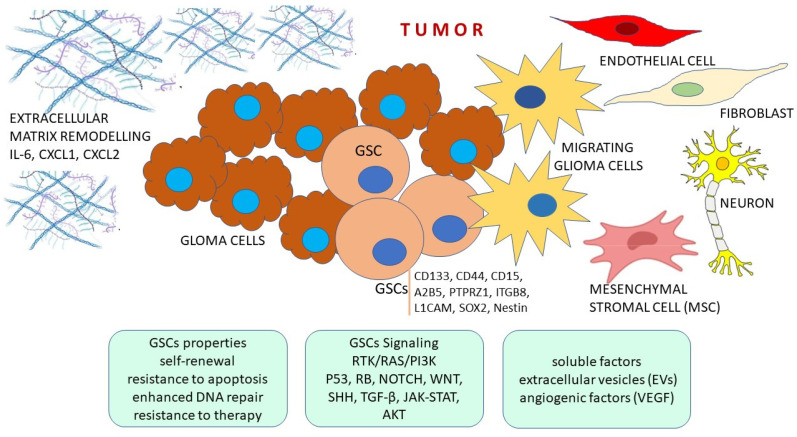
GSCs are responsible for tumor plasticity and are influenced by genetic drivers. GSCs also display greater migratory abilities. Glioblastoma consists of heterogeneous cellular population. Tumor cells, including GSCs and glioma cells, interact with various types of cells in tumor. Distinct characteristic of GSC cells are shown, for example the capacity for self-renewal, proliferation and differentiation into diverse cell types within tumor mass. Versatile soluble factors are secreted by GSCs to recruit and activate stromal cells and reorganize the ECM, as well as to promote angiogenesis, metastasis, hypoxia, immune evasion and tumor progression. Alterations include genetic alterations, signaling pathways, vascularization and metabolism. Glioma stem cells (GSCs) are influenced by these factors, but also reciprocally influence TME. GSCs markers are indicated.

Stem cells are able to differentiate into different cell types and exhibit great plasticity. Similar features can be found in cancer stem cells (CSCs). Cells that are able to self-renew and show high invasiveness and metastatic features have been discovered in many cancer types, including hematologic malignancies, solid tumors, breast cancer, colon cancer and melanomas, among others. Although they display similarities to normal stem cells, they are not identical and have distinct features that make them capable of tumorigenesis driven by few tumorigenic genetic mutations. In gliomas, a CSC subpopulation with tumor-initiating properties has also been discovered [35]. Those cells have been named glioma stem cells (GSCs), or glioma cancer stem cells (GCSCs) and they also show the ability for asymmetric division, self-renewal and can give rise to various cell types (Table 1 and Figure 1). Both autocrine and paracrine signals from the surrounding stroma are involved in their differentiation and maintenance. They are responsible for tumor plasticity and are influenced by genetic drivers. On top of that, GSCs also display greater migratory abilities through the process known as the epithelial–mesenchymal transition (EMT) [36]. EMT occurs during invasion and metastasis. In this transition, the nonmotile cells lose their cell polarity and cell–cell adhesion, and acquire further molecular changes that enable them to reach a mesenchymal phenotype with marked migratory potential, changed extracellular matrix and cytoskeleton, invasive behavior as well as resistance to apoptosis. The brain has a specific and ample extracellular matrix abundant in glycoproteins, proteoglycans and glycosaminoglycans. Recent data show that proteoglycans such as heparan sulfate and hyaluronic acid are accumulated in definite areas and contribute to formation of stem cell and vascular niches [37]. Extracellular matrix components assist in a correlated activity between the tumor cells and surround stroma. 

Further properties shared between normal stem cells and GSCs are neoangiogenesis, resistance to apoptosis and lineage determinization, while recovery from conventional therapeutic attacks, enhanced capacity of DNA repair, metastasis, xenobiotics expelling and finding reactive oxygen species are attributed to GSCs. 

The origins of glioma are still controversial. It is believed that progenitor cells of neural and oligodendrocyte lineage are plausible cellular origin for glioma development. In normal circumstances, neural stem cells/progenitor cells that will give rise to neuron/glial lineage are localized in a region called the subventricular zone (SVZ) [38]. Studies indicate the localization of GSCs to a vascular niche and show that they arise from cells of the subventricular zone (SVZ) or from differentiated glioma cells [34]. Distinct cells in the tumor reflect the developmental state of glioblastoma cells. Along with genetic, epigenetic and environmental causes of heterogeneity, there is also a developmental cause [39].

Along their way of transformation and proliferation, GSCs start from proneural phenotypes to invasive mesenchymal ones. However, it is their migratory potential that determines the generation of malignant gliomas in distinct brain regions [40].

The abundance of signals that regulate GCSs leads to difficulties in studying this subpopulation of cancer stem cells. In spite of the fact that a series of biomarkers associated with cellular stemness in gliomas have been identified in GSCs, the results are still controversial and cannot be used. The primary markers of GSCs are CD133, CD44, CD15, SOX2, OCT-4 and Nestin [38]. However, these markers are not sufficiently specific. For example, CD133+ has also been found in pilocytic astrocytomas, gangliogliomas and medulloblastomas. Unfortunately, no consensus on the markers of glioma stem cells has been established yet because the results of different studies were controversial. Of note is that Nestin, Sox2, CD44 and CD133 are displayed both in NSCs and in GSC [41]. Moreover, there is evidence of genetic heterogeneity in the subpopulation of glioblastoma cells expressing different sets of molecular markers since cancer stem cells show high transcriptomic instability allowing them to proceed along various cell fates in response to extrinsic and intrinsic stimuli [42]. However, novel techniques such as single-cell sequencing (scRNA-seq) are showing promise for molecular characterization of each specific cell that transition along differentiating paths. Several novel papers showed four cellular states of glioma cells: (1) OPC-like, (2) NPC-like, (3) astrocyte (AC)-like and (4) mesenchymal (MES)-like. Thus, knowledge about the formation of GSCs is essential for explaining the source of heterogeneity in gliomas [38]. Additionally, distinct GSC molecular profiles are identified in the different GBM subtypes. For instance, the mesenchymal subtype shows low CD133 expression levels and high levels of CD44, YKL40, BMI1, ALDH1A3, TWIST1, SNAI1-2, TGFB1, STAT3 and CD248 GSC markers, whereas the proneural subtype is characterized with the high expression of GSCs markers CD133, OLIG2, SOX2 and EZH2.

It is necessary to also address the DNA-methylation profile of GSCs. DNA-methylation changes can be used as biomarkers at different stages of the tumor as they are acquired somatically in the course of tumor progression. Methylome refers to the complete set of DNA methylation modifications of a cell’s genome, but relatively little is known about methylation regulatory mechanisms and how precisely the epigenetic changes influence tumorigenesis [37]. In brain tumors, Capper et al. (2018) [43] determined nearly 100 different entities across different age groups. Gargini et al. (2020) [37] indicated the differences between the methylation patterns of IDHmut and IDHwt gliomas which is in accordance with the similarities between IDHwt astrocytomas and GBMs. Furthermore, they emphasize the difference between pediatric versus adult gliomas.

Cancer cells are characterized with metabolic plasticity, which has been shown to play a pivotal role in drug resistance. Metabolic alterations in glioblastomas including GSCs have been found [44,45]. Alterations occur in glycolysis, mitochondrial oxidative phosphorylation (OXPHOS), the pentose phosphate pathway (PPP), lipids, amino acids and nucleotides metabolism. Proliferating glioblastomas predominantly use aerobic glycolysis (Warburg effect) for energy production. Contrastingly, GSCs, whether they are isocitrate dehydrogenase 1 wild-type (IDH1wt) or isocitrate dehydrogenase 1 mutant type (IDH1mt), mainly use OXPHOS and lipid metabolism [46]. Metabolic therapy in treating glioblastomas, especially for the elimination of glioma stem cells (GSCs), has been recognized as an encouraging approach by reason of the metabolic alterations present in cancer cells [47]. GSCs are situated in hypoxic microenvironments and retain slowly proliferating states which protect them from chemotherapy and radiotherapy [48]. It is known that many proteins act as therapeutic targets to reduce OXPHOS, primarily in GSCs such as glycerol-3-phosphate dehydrogenase (GPDI), insulin-like growth factor 2 mRNA-binding protein 2 (IGF2BP2/IMP2), oncostatin M and translocator protein (TSPO) (van Noorden et al., 2021). Recently, several studies have demonstrated hopeful inhibitors of mitochondrial activity in GSCs which may lead in GSCs citotoxicity [49,50,51]. Mudassar et al. (2020) [52] suggest inhibiting OXPHOS by increasing the low oxygen levels in hypoxic microenvironment of GSCs to sensitize GSCs to irradiation by repurposing antimalaria drugs. Kuramoto et al. (2020) [53] described the cytotoxic effect of verteporfin specifically to GSCs and not to differentiated glioblastoma cells or normal cells. Verteporfin was approved by the Food and Drug Administration (FDA) for macular degeneration and it inhibits OXPHOS activity. One study found that mesenchymal and proneural GSCs are different in both their metabolic pathways and their response to metabolic therapies [54]. Mesenchymal GSCs metabolized glucose through glycolysis and were less responsive to metformin, whereas proneural GSCs metabolized glucose through the PPP and were more responsive to metformin, meaning that they were less invasive. Metformin reduces cell biogenesis, proliferation and migration, while increasing apoptosis in GSCs [55]. A recent study showed the obvious detrimental impact of temozolomide (TMZ) with metabolism inhibitors, gossypol and phenformin on energy production, stemness and invasiveness in GSC lines compared to TMZ monotherapy or gossypol and phenformin dual therapy [56]. 

Mitochondria are the metabolic intersection for many metabolic pathways. Those organelles can be transferred to cancer cells and modify the energy metabolism of the target cell. The transfer can happen in different ways, however it often occurs through tunneling nanotubes (TNTs or TnTs). The modification of energy metabolism leads to OXPHOS increases at the expense of glycolysis [57]. Such metabolic changes enhance proliferation and migration of cancer cells. Moreover, the acquisition of cancer drug resistance was also associated to TNT-mediated mitochondria transfer [58]. This points to mitochondria as targets for therapeutic interventions. Mitochondria can be targeted directly by so called ‘mitocans’, which are different small bioactive molecules such as paclitaxel, etoposide, vinorelbine, ceramide, lonidamine or betulinic acid. They act on cytochrome C release and consequent apoptosis of cancer cells. Antibiotics are also specific candidates for mitochondria targeting. Another approach is targeting ROS, which is promising in cell death induction.

Ion channels play important roles in many cellular functions. Signaling through calcium channels has been responsible for stem cell proliferation and migration, and recent transcriptomic studies have highlighted the fact that calcium pathways predominate in glioblastoma stem cells. GSCs are more sensitive to Ca^2+^ oscillations compared to more mature cells [59]. Furthermore, Terrié et al. (2021) [60] showed that GSCs express store-operated channels (SOC), which are one of the major pathways for Ca^2+^ entry and whose pharmacological inhibition reduces GSCs proliferation and self-renewal. One of the most important regulators of calcium signaling is calcium/calmodulin (CaM)-dependent protein kinase II (CaMKII). New data indicate a prominent role of CaMKII kinaze in the survival, proliferation and maintenance of cancer stem cells [61]. Targeting CaMKIIγ with combined inhibitors increased GSCs lethality by downregulating the CaM/CaMKII/c-Met signaling pathway [61,62]. Furthermore, dual inhibition strongly suppressed the expression of several GSC markers, such as CD133, integrinɑ6, ALDH1A1, Nanog, Sox2 and Oct4, that play key roles in GSCs maintenance and drug resistance. Co-treatment with berbamine and ArcA notably downregulated the expression levels of cell cycle regulatory proteins by firmly inactivating the CaMKII-mediated STAT3/AKT/ERK1/2 signaling pathway in GSCs.

## 3. Tumor Microenvironment

**Figure 2:**
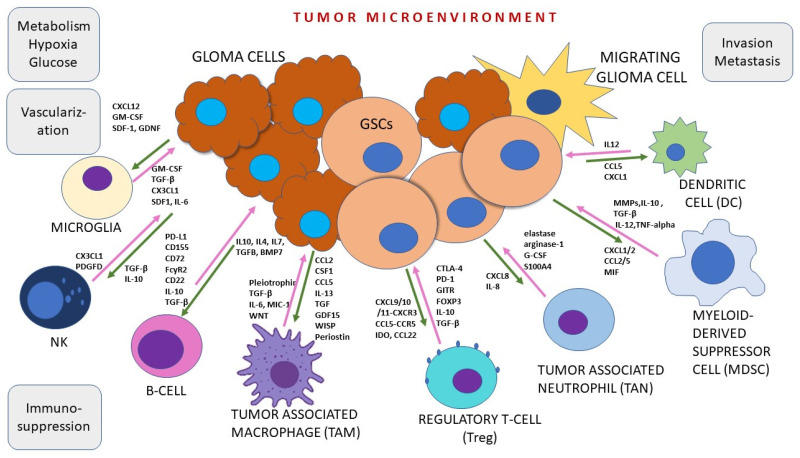
GSCs are plastic tumor cells that reside in vasculature-rich surrounding and interact with distinct immune system cells and their molecules. Immune microenvironment as a segment of tumor microenvironment consists primarily of brain-resident microglia and infiltrating monocytes. Glioma stem cells are influenced by TME factors, but also influence TME. Reciprocal interactions between GSCs and distinct immune cell subsets is shown. Abundance of signals warrant complex interactions in TME. (TGF-β, transforming growth factor-β; GDNF, glial-derived neurotrophic factor; TNF-α, tumor necrosis-α; GM-CSF, granulocyte-macrophage colony-stimulating factor; VEGF, vascular endothelial growth factor; CX3CL1, chemokine (C-X3-C motif) ligand 1; CXCL12, C-X-C motif chemokine 12; BMP7, bone morphogenetic protein 7; SDF-1, stromal cell-derived factor 1 (SDF-1), also known as C-X-C motif chemokine 12 (CXCL12); IL-6, interleukin-6; IL-10, interleukin-10; MIC-1, macrophage inhibitory cytokine 1; MIF, Macrophage migration inhibitory factor CCL2, C-C motif chemokine ligand 2; CTLA-4, Cytotoxic T-lymphocyte-associated protein 4; GITR, Glucocorticoid-Induced TNFR-Related protein; PD-L1, Programmed death-ligand 1; IDO, Indoleamine 2,3-dioxygenase; MMPs, Matrix metalloproteinases; S100A4; NK, natural killer cells).

Another point worth discussing is the adaptation of glioma cancer stem cells to the highly competitive brain environment. The environment is hypoxic and immunosuppressive with aberrant blood brain barrier (BBB) integrity and changed vasculature. GSCs engage in a synergistic relationship with the surrounding tumor microenvironment to promote tumor progression and therapy resistance [63]. It has become apparent that CSCs have features to form their own self-advantageous environmental niche [37,64] by taking over and remotely controlling the host inflammatory and hematopoietic cells (Figure 2). Equally important is the communication among different cellular types in each glioma microenvironment niche [65]. GSCs are plastic tumor cells that reside in vasculature-rich surroundings in stromal regions and interact with distinct immune system cells and their molecules. The multiple interacting cellular networks consist primarily of brain-resident microglia and infiltrating monocytes [38]. It has become clear that the aggressive nature of glioblastoma can be attributed to the specific soil in which it resides where the aggressive behavior of this tumor is fueled by the specificity of the brain tissue. The full list of immune cells in the glioma microenvironment consists of myeloid-derived suppressor cells, T cell subset called T-regs, dendritic cells and neutrophils (Figure 2), along with microglia and macrophages. Microglia are crucial residential innate immune cells of the brain. In several investigations using mouse models of glioma, microglia were found mainly at the tumor margins promoting proliferation, infiltration and stemness [66,67]. It has also been demonstrated that the silencing of microglial function results in reduced tumor proliferation.

Spatial omics provide further understanding of complex cellular interactions by accounting for endogenous tissue architecture and how close specific cells are to each other [68]. Spatially related functions of glioma cells primarily include progression and invasion. Microglia-derived tumor-associated macrophages (MDMs) are observed in all areas of tumor mass with an extremely high distribution near vessels, while resident microglia are often confined to tumor border areas and are absent from the tumor core in GBM [69]. 

According to novel literary findings, microglia can produce and release distinct factors that stimulate glioma proliferation and invasion. One of the primary factors is the epidermal growth factor (EGF). There is also a cellular prion protein ligand stress-inducible protein 1 (STI1). TGF-β is also commonly released from microglia. It has been demonstrated that the first identified microglial chemoattractant was monocyte chemoattractant protein-1 (MCP-1), also known as CCL2 (C-C motif ligand 2), that acts through the CCL2 receptor (CCR2). CCR2 is expressed on microglia [70] and can trigger the release of IL-6 from microglia, which in turn promotes the invasiveness of glioma cells [66]. Toll-like receptor 2 (TLR2) is also important, as it induces MT1-MMP (membrane type 1–matrix metalloproteinase) upregulation. MT1-MMP is upregulated in microglia when exposed to glioma cells. However, all microglia factor producing activity begins with CSF-1, a factor that is chemoattractant for microglia and is constitutively released by tumor cells [71,72,73].

The second major cellular component in the glioma niche represent macrophages. Monocytes that pass the BBB and infiltrate the tumor mass are called tumor-associated macrophages (TAMs). They account for approximately 30% of tumor mass in human GBM. The study conducted on newly diagnosed GBM, recurrent GBM and mouse GL261 models by Pombo Antunes et al. (2021) [74] using the microglial fate-mapping system showed similarities and differences in TAM distribution. It has also been shown that microglia-derived TAMs or MDMs extracted from tumors carry self-renewing characteristics. When stimulated by cancer cells including GSCs, TAMs can release a variety of cytokines and growth factors. For example, TAMs increase the production of anti-inflammatory molecules, namely transforming growth factor β (TGF-β), ARG1 and IL-10. In addition, they produce factors that support angiogenesis and tissue remodeling, VEGF, MMP2, MMP9 and MT1-MMP. Furthermore, TAMs can synthesize pro-inflammatory molecules such as IL1-β, TNF-α and CXCL10 [66,75]. 

In addition, the infiltration of TAMs in tumors was mediated by numerous chemotactic factors produced by tumor cells including C-C motif ligand 2 (CCL2), CX3C chemokine ligand 1 (CX3CL1), stromal cell-derived factor-1 (SDF-1), colony-stimulating factor-1 (CSF-1) and periostin (POSTN), among many other molecules augmented in present studies [76]. Chemoattractants for microglia released by macrophages include Hepatocyte growth factor/scatter factor released by glioma cells [77], while CXCL12 (SDF-1) is also a potent microglia and macrophage recruiting molecule, especially for attracting TAMs to hypoxic areas. Zhang et al. have demonstrated that Programmed Cell Death 10 mediates CXCL2-CXCR2 signaling in recruitment of TAMs in glioblastoma [78,79]. 

Although difficult, it is important to distinguish microglia from macrophages. This can be achieved using CD45 antibodies that recognize CD45, a hematopoietic transmembrane tyrosine phosphatase that is present on the surface of all hematopoietic cells. Resident microglia express low levels of CD45, while macrophages of hematopoietic origin express high levels of this molecule [80]. 

Furthermore, vasculature is also an important aspect of glioma microenvironment. High-grade gliomas are highly vascularized tumors characterized by the overexpression of proangiogenic factors [81]. Complex interactions between tumor cells, and their immune and vascular niches, propel glioma malignancy and determine their response to therapy. Endothelium of blood vessels is exposed to signaling that promotes vascularization through a wide variety of angiogenic molecules and cytokines. Simultaneously, tumor leukocytes are affected by them. Notably, the oncogenic signaling works in both directions. GSCs influence the formation and modification of new tumor vessels. In return, vascular-niche-specific expression provides the metabolic milieu and the appropriate extrinsic signaling that is stimulative for the maintenance of the tumor stem cells. Lately, a similar bidirectional crosstalk has been established for other tumor ecosystems. In that respect, GSCs crosstalk signals to hypoxic or immune ecosystems [82]. 

It is known that GSCs influence immune suppression by preventing immune cells from sufficient uptake of glucose and oxygen. Furthermore, they inhibit T cell proliferation and cytotoxic T cell activation. They also secrete factors IL-10 and TGFβ, which interfere with the tumor-killing function of macrophages [83].

Neuronal–GBM cell interactions have recently emerged as contributors of glioblastoma invasion and progression. Such neuron-cancer synapses induce the release of neuroligin-3, dopamine and brain-derived neurotrophic factor (BDNF). Moreover, local neuronal excitability is also induced by GBM cells [84].

Tumor microenvironment is a complex arena where different signals, trafficking of secreted factors and extracellular matrix components take place. Communication can happen through multiple ways: secreted soluble factors such as chemokines and cytokines, extracellular vesicles (EVs) and direct cell–cell contacts such as gap junctions and tunneling nanotubes (TNTs or TnTs). TNTs allow for the intercellular exchange of vesicles, viruses, miRNAs, proteins, mitochondria and lysosomes as well as Ca^2+^ ions [84]. Tunneling nanotubes are long nanometric structures that range from 20 to 700 nm in width, and are made of thin plasma membrane that allow the intercellular exchanges of materials and signals between distant cells [85]. TnTs can mediate both heterocellular and homocellular interactions. Novel research indicates that many different cell types that build CNS communicate with each other through TNTs bridges [86]. Along with roles in normal brain physiology, tunneling nanotubes (TNTs) are found to be involved in glioblastoma (GBM) intercellular crosstalk. Those long channels connect both nearby and distant cells, contributing to glioblastoma malignant phenotype and progression. TNTs provide an explanation for the heterogeneity in gliomas and their resistance to therapy [87]. Simone et al. (2023) [88] demonstrated that the glial fibrillary acidic protein (GFAP) plays a structural and functional role in TNT-mediated crosstalk between cells promoting glioblastoma progression. Oxidative stress, cytotoxic treatments and ionizing radiation all contribute to TNT communication in GBM, since enzymes, organelles and other cargo and signals travel from resistant to sensitive cells [87]. Particularly important is the TNT-mediated transfer of mitochondria, as in glioblastoma the arrival of mitochondria can transition non-tumor astrocytes to tumor-like metabolism and hypoxia conditions [89,90]. However, the full molecular composition of TNTs still needs to be elucidated. Of particular interest for understanding cellular communication in GBM are EVs. They have emerged as critical regulators of communication between cancer and the surrounding cells [91]. Packed with different substances as cargo, EVs can contain RNA, DNA, proteins, enzymes, transcription factors, metabolites, viruses and even mitochondria. According to their size, cargo, biogenesis and function, EVs are divided into exosomes, microvesicles, apoptotic bodies and large oncosomes [84]. Glioblastoma cells have been shown to release those nano-sized vesicles in order to influence their environment. Exosomes (50–200 nm) and microvesicles (>100 nm–1 µm), have recently been observed contributing towards intercellular communication within the tumor niche [92]. In glioma specifically, exosomes carry miRNAs and lncRNAs [93] that contribute to tumor evolution. Originating from multivesicular bodies (MVBs) by double invagination of the plasma membrane, exosomes are able to traverse natural BBB. Glioma cells usually exhibit higher EVs release compared with healthy cells. Cargo composition also differs from normal glial cells. Oncogenic effects that tumor-derived EVs cause are altered metabolism, the promotion of angiogenesis, invasion and immune suppression. There is also a class of extracellular vesicles formed by endocytosis. These are small extracellular secretory vesicles (SEVs) originating from the endosome. SEVs that are secreted by glioma tumor cells are called tumor-derived exosomes (TEXs), and they have a vital role in tumor progression and metastasis as they represent a major communication mechanism between tumor cells and tumor microenvironment. However, the application of TEXs in the diagnosis and treatment of glioma is still in the nascent stages [94,95].

Furthermore, cancer cells develop relative resistance to treatment through the deregulation of endoplasmic reticulum (ER) homeostasis. Various pathological conditions including hypoxia, glucose deprivation and chemotherapy can disrupt the protein folding capacity of the ER leading to ER stress. The production of misfolded proteins hinders protein homeostasis and normal cellular functions. Quality control mechanisms named the unfolded protein response (UPR) are triggered to restore homeostasis [96]. However, despite its adaptive role, it has been demonstrated that UPR response has promotive role for glioma progression. It has been demonstrated that UPR is involved in regulation of stem cell properties, but the mechanisms have not yet been defined.

As far as lysosomes’ role in glioblastoma is addressed, it is important to highlight [97] that, for their own benefit, glioblastoma cells modify lysosomes both qualitatively and quantitatively. Results of many novel studies suggest that tight regulation of the lysosomes is needed for sustaining GSC stemness [98]. It has been shown that lysosomal homeostasis impairment was efficient in stopping GSC growth. Therefore, lysosomes also emerge as appealing targets for glioblastoma therapy.

## 4. Signaling Pathways in Glioma Cancer Stem Cells

Malfunctioning of signaling pathways is responsible for glioma formation and development. The interactions among cellular signaling pathways regulate and influence their activity forming signaling networks of mutual communications. Deregulation of complex signaling mechanisms is a culprit in glioma initiation and progression. Although it is still impossible to define the exact number of changes and the chronology of their occurrence during gliomagenesis, a long-scale study conducted by TCGA revealed responsible signaling participants. Thus, key genes that are most frequently altered fall into three deregulated oncogenic pathways: (1) RTK/RAS/PI3K, (2) TP53 and (3) RB (Figure 1).

It is important to note that in GSCs, pathways specifically associated with the maintenance of the stem-like phenotype are critically involved. The molecular components of these pathways increase survival and make GSCs more resistant to cytotoxic therapies. The following pathways play important roles in GSC: HIF1α, NOTCH, WNT, SHH, TGF-β, STAT3, AKT and EGFR. The pathways are involved in GSC growth, proliferation, migration and invasion [99,100] (Table 2). 

Epidermal growth factor receptor (EGFR) pathway is involved in the regulation of proliferation, migration and differentiation. Aberrant signaling of EGFR have commonly been detected in GBM. In GCSCs gene amplification, rearrangements of EGFR were found and EGFR activity was raised. Such aberrant signaling promotes self-renewal and tumorigenicity, thereby causing resistance to chemotherapy and radiotherapy. EGFR is an important factor that is often upregulated and overexpressed in GBM, and is also frequently mutated. Changes of EGFR subsequently affect many downstream signaling pathways. The AKT pathway is one of them and is often abnormally activated during the development of GBM [101]. A great number of studies on Akt pathway, which in its expanded version includes phosphatidylinositol 3-kinase (PI3K)/Akt/rapamycin-sensitive mTOR-complex (mTOR) signaling [102] have reported the frequency of mutations and copy number aberrations of its components in glioblastoma to be around 88% [102,103]. Therefore, the inhibition of AKT activation is a beneficial strategy for glioma treatment. 

In addition, many studies have shown that TGFβ (transforming growth factor β) is vital for cancer stem cell biology. It is especially vital for GBM, where elevated levels of TGFβ activity correlate to poor patient outcome. TGFβ pushes proliferation of GBM cells, acting through platelet-derived growth factor-B (PDGFB), NF-κB (nuclear factor of κ light polypeptide gene enhancer in B-cells) and nodal. Furthermore, with the help of Smad2/3, which is its signaling mediator, TGFβ promotes the expression of Sox4 and LIF (the cytokine leukaemia inhibitory factor). In succession, the self-renewal capacity of the GSCs is increased by the expression of the stem cell transcription factor Sox2. Therefore, the GSC tumor-initiating potential is ensured [104,105].

Signaling involved in glioma cancer stem cells is hypoxia pathway. Two main molecules manifesting in the response to hypoxia in glioma are hypoxia-inducible factor-1α (HIF1α) and hypoxia-inducible factor-2α (HIF2α) [106]. Qiu et al., (2020) [107] indicates that those factors lead to chemoresistance of GSC by maintaining stemness. The outcome of HIF1α/HIF2α-Sox2 signaling in a hypoxic microenvironment is the dedifferentiation of differentiated glioma cells leading to the formation of glioma stem cells. Such dedifferentiation led to glioma cell chemoresistance. The study by Wang et al. (2022) [108] demonstrated that both HIF1α and HIF2α, genes that are functioning upstream of Sox2, regulated the malignant progression of glioma through dedifferentiation. 

The maintenance and development of the central nervous system depend on the proper functioning of Notch signaling [99]. Notch is another highly preserved signaling pathway which is involved in many cell fate decisions such as cell proliferation, differentiation and neurogenesis. Furthermore, it is also implicated in malignant transformation. Notch pathway is activated when sending and receiving cells are present. Sending cells provide ligand proteins (Delta-like and Jagged ligands), while receiving cells contain single-pass transmembrane Notch receptors (Notch1-4) composed of functional extracellular (NECD), transmembrane (TM) and intracellular (NICD) domains. Upon ligand-receptor crosstalk, the NECD is cleaved away by proteins ADAM/TACE. A highly active Notch signal has emerged as a crucial player in GSCs. Events that lead to the activation of the canonical Notch target genes in the signal-receiving cell, include the cleavage of NICD from the TM by enzyme γ-secretase. Thus, NICD can be translocated to the nucleus where it associates with the CSL transcription factor complex, needed for target gene activation.

Regulation of Notch signaling pathway has been implicated at multiple levels of cancer biology, encompassing neoplastic cell growth, cancer stem cell maintenance, angiogenesis and metastasis [109]. A key component within the signaling pathway—NICD—has been found to accumulate in the nucleus as the result of Notch receptor mutations. This translocation modulates gene expression in several cancer types [110]. Another study [111] on CXCR4 upregulation has identified Notch pathways as a promoter of glioma stem cell self-renewal and invasiveness.

Recent investigations have highlighted the sonic hedgehog (SHH) signaling pathway as a crucial player in the proliferation and cell fate specification of stem cells [112,113]. The GLI gene-mediated hedgehog (Hh) pathway plays an essential role in different types of brain cancer, including lower-grade glioma (LGG), glioblastomas (GBM) and medulloblastomas (MB). Stemness-related factors of GSC, namely SOX2, SOX9, POU5F1 and NANOG demonstrate correlation to GLI genes. The expression of transcription factors, GLI2 and GLI3 is increased in the aforementioned tumors with poor patient survival [113]. A study on patient-derived GSC cultures by Liu and collaborators (2022) [114] indicated that Islet 1 (ISL1), a member of the homeodomain transcription factors, knockdown decreased the proliferation and promoted the apoptosis of human-derived GSC. Moreover, ISL1 influenced downstream expression of GLI1 by acting on SHH. However, the inhibitory effect of ISL1 knockdown on GSC growth and tumorigenesis was alleviated by the application of recombinant SHH. 

Transcription factors of the signal transducer and activator of transcription (STAT) protein family are implicated in various aspects of cellular life, including apoptosis, proliferation, differentiation and immunity. Once activated by membrane receptor-associated Janus kinases (JAKs), STATs promote angiogenesis, tumor cell survival and immunosuppression [69] in primary tumors. JAKs are activated by binding inflammatory cytokines or growth factors to the receptors. The subsequent step performed by JAKs involves phosphorylation of a target tyrosine residue within the STAT protein. Specifically, STAT3 overexpression correlates with a high glioma grade, poor prognosis and lower survival of glioblastoma patients [115]. 

It has been well-established that Wnt signaling is responsible for tumorigenesis and maintenance of various human cancers [1,116,117,118,119]. The main signaling effector molecule of the pathway is beta-catenin, whose oscillation in cellular levels regulates the activity of the pathway [120,121]. Continuous elimination of beta-catenin from the cell prevents it from reaching the nucleus, thereby rendering the pathway inactive and repressing transcription of Wnt target genes [122,123]. 

Results of previous research on Wnt signaling clearly demonstrates the involvement of Wnt pathway in the biology of gliomas. Our investigations have identified the patterns and correlations of genetic and protein changes of Wnt molecular components in both lower- and higher-grade gliomas. Clinical and demographic parameters were correlated and associated to genetic findings, as well as protein subcellular localizations. Our results show that Wnt pathway is activated. The investigation on three human Disheveled genes (DVL1, DVL2 and DVL3) evidenced on their association to transcription factors LEF1 and TCF1 influencing their important roles in glial branch of brain tumors [124]. Additional findings indicated that transcription factors of the Wnt pathway, TCF1 and LEF1, were upregulated in malignant high-grade gliomas [125]. The investigation on secreted frizzled-related protein 3 (SFRP3) showed its modulation of the Wnt signaling cascade [126], and its expression levels suggested its dual role in Wnt signaling depending on the context. Our next findings on DVL3 and SFRP3 expressions and localizations [127] showed that the expression levels of the two proteins were not correlated, but that the category of strong expression of DVL3 more often localizes the protein in the nucleus. The research on the secreted frizzled related protein 1 (SFRP1) promoter hypermethylation [128] showed that this gene was epigenetically silenced in glioblastomas when compared to lower astrocytoma grades, suggesting its involvement in tumor progression. Our in silico investigation on genes that are participants of EGFR-PI3K-AKT-mTOR signaling using data from the publicly available cBioPortal database showed different numbers of copy number aberration of PTEN (76%), PIK3AP1 and CHUK (75% each), EGFR (74%), AKT2 (39%), AKT1 (32%), AKT3 (19%) and GSK3β (18%) in the 751 samples of diffuse glioma as well as their mRNA expression levels [129].

It has been demonstrated that activation of the Wnt signaling is important in the maintenance and proliferation of GSC, as well as in glioma local invasiveness and infiltrativeness, and chemoresistance and radioresistance [130]. Specifically, the primary pathway associated with the process of glioma cell invasion is the Wnt pathway. Du et al. (2020) [131] demonstrated that the expression of β-catenin was significantly higher in glioma compared to normal tissues. In addition, higher expressions of Frizzled2, Wnt2, β-Catenin and Wnt5a were observed in gliomas [132]. When silencing Wnt2 and β-catenin by siRNA in the human glioma U251 cells, proliferation and invasion were inhibited and apoptotic cell death was induced [132]. The intracranial transplantation model of glioblastoma mice demonstrated that the inhibition of Wnt5a activity could prevent the process of brain invasion and enhance the survival rate of the host [99]. Of further interest is the fact that Wnt signaling can regulate O6-Methylguanine-DNA methyltransferase. Members of WNT pathway (frizzled receptors (FZD2/7), β-catenin, TCF7L1/2, and LEF1 transcription factors, E-cadherin (CDH1), phospholipase C gamma (PLCG1), calmodulins (CALM1/2/3), calcineurin (PPP3CA, PPP3CB, and PPP3CC), and nuclear factor of activated T cells (NFATC4)) are associated with the mesenchymal GBM subtype and are characterized as specific prognostic markers [119]. The Wnt pathway is very much involved in EMT, thus participating in glioma plasticity and migration [133]. Malfunctioning of Wnt pathway was noted in the formation and maintenance of GSCs [134,135,136]. Not only ligands and receptors, but also negative and positive regulators of Wnt signaling were overexpressed, and controlled EMT and tumor microenvironment communication [137,138].

## 5. Potential Therapeutic Approaches

GSC cellular population can maintain oncogenicity and represent the main reason why the ongoing therapeutic efforts are inefficient. Therefore, a great effort is under way to find ways to eliminate or neutralize them (Table 2). Many different treatment strategies at targeting GCSs are currently investigated, including modulation of the tumor microenvironment, posttranscriptional regulation, epigenetic modulation and immunotherapy [34,139].

Targeting the tumor microenvironment, especially microglia and macrophages, is one of the intriguing possibilities as an adjuvant therapy for difficult-to-manage gliomas. Multiple strategies are being investigated, and some are tailored to block the immediate interactions among cells. Others attempt to neutralize secreted and circulating factors that support both immune cells and GSCs [140]. The approach that holds promise to improve disease outcome is the disruption of communication between GSCs and immune cells. This would maximize benefits since it would improve recognition by cytotoxic T cells. Mass cytometry disclosed that 72.6% of the leukocytes in the tumor microenvironment are macrophages, most of which expressed all kinds of immunosuppressive factors. Taken together, these finding demonstrate that immune suppressive macrophages are indispensable for attenuation of the T-cell response. 

It has been demonstrated that GSCs escape recognition by the immune system owing to the expression of checkpoint inhibitors, PD-L1, CD70, A2aR and TDO, but also to the decrease of major histocompatibility complex class I (MHC-1) molecules [141,142]. Therefore, several clinical trials targeting immune checkpoints are under way. Noteworthy is the fact that a phase III clinical trial that compared the effects of two monoclonal antibodies—PD1 (nivolumab) and VEGFA (bevacizumab)—unveiled that patients with recurrent glioblastoma did not benefit from nivolumab treatment (CheckMate-143) [143]. A phase III clinical trial ascertained that patients with de novo O-6-methylguanine-DNA methyltransferase (MGMT) unmethylated glioblastoma that received nivolumab plus radiotherapy did not benefit from nivolumab (CheckMate-498) when compared to standard chemoradiotherapy [144]. 

Tumor immunotherapy has received a significant amount of research attention. Seeing as numerous factors affect brain immunity, current immunotherapy strategies necessitate further improvement prior to application in high-grade gliomas [38]. 

Targeting metabolic pathways that determine the interplay between GSCs and immune cells has the potential to improve current therapies, primarily by making the tumor microenvironment more responsive to immunotherapies [83].

Nanobodies are novel tools that target specific molecules and inhibit their effect. They are variable domains of the functional heavy-chain antibodies, size 12–14 kDa, naturally occurring in the serum of Camelidae species [145]. They are soluble, have strong affinity for their targets, have nanometre dimensions, show low immunogenic risk and are especially suited for treating gliomas since, due to their nanometer dimensions, they are able to pass BBB [146].

TNTs can be exploited for new strategies of drug delivery. Delivering cures through those long bridges that can reach into the glioblastoma niche where GSC reside represents powerful anti-cancer strategies. Preventing mitochondria transfer could represent particularly efficient approach.

The presence of EVs opens an entirely new perspective on drug delivery in GBM. For example, exosomes have an excellent drug-carrying capacity since they are non-toxic and BBB penetration is readily achievable. Recent study engaged paclitaxel and doxorubicin loaded exosomes isolated from brain endothelial cells to treat GBM [147]. McDonald et al. (2024) [148] explored several microRNAs packed into engineered exosomes (eExos) as novel therapeutics for GBM. Glioma stem cell-derived exosomes were also recently investigated [149] and were able to inhibit glioblastoma development. Thus, exosomes are regarded as promising candidates for brain disease therapeutics and diagnoses, and many exosome-based strategies currently entered different clinic trial stages. Since UPR is involved in the pathophysiology of GBM, it may provide novel therapeutic targets. For example, UPR inhibition was shown to sensitize for temozolomide. Peñaranda-Fajardo et al. (2019) [96] propose that ER stress induction is beneficial for GMB patients since it suppresses self-renewal potential of GSCs.

Notably, there is also an aspect of ER-mitochondrial contact sites and homeostasis [150,151]. ER mitochondrial homeostasis is essential for regulation of GSCs glucose metabolism and survival. Disruption of ER homeostasis using ER stress inducers or inhibition of ER mitochondrial contact sites using the Grp75 inhibitor MKT 077 resulted in cytotoxicity of glioma cells and loss of stemness. Moreover, the effect of temozolomide was potentiated. Targeting the mitochondrial contacts with the ER could be an innovative strategy to deplete the cancer stem cell compartment to successfully treat glioblastoma [150].

## 6. Conclusions

Glioblastoma is characterized by high mortality and is associated with discouraging prognosis as a result of its aggressive and infiltrative nature, late diagnosis and treatment resistance. GSCs are responsible for tumor plasticity and are influenced by genetic drivers. GSCs also display greater migratory abilities. Due to the complexity of glioma stem cells that do not terminally differentiate, further research is needed to fully understand their role in gliomas. Many different treatment strategies at targeting GCSs are currently investigated.

## Figures and Tables

**Table 1:** Signaling pathways involved in GSC and frequency of gene alterations in gliomas, from cBioPortal—a publicly available database for tumor genomics and transcriptomics (https://www.cbioportal.org/, accessed on 1 April 2024).

Signaling Pathway	Gene for Signaling Component	Frequency of Gene Alteration *
RTK/RAS/PI3K	EGFR,	29.04%
	PDGFRA,	8.89%
	MET,	4.15%
	FGFR2,	2.27%
	FGFR3,	2.81%
	PTEN	41.82%
p53	TP53,	43.47%
	MDM1,	2.39%
	MDM2,	5.84%
	MDM4,	4.46%
	CDKN2A	33.02%
RB	RB1,	11,27%
	CDK4,	10.78%
	CDK6	2.68%
NOTCH	NOTCH1 (NICD),	5.87%
	NOTCH2	2.47%
	NOTCH3	3.12%
	CXCR4	0.54%
WNT	CTNNB1,	0.93%
	LEF1,	0.54%
	TCF1 (HNF1A)	1.4%
	DVL1,	1.74%
	DVL2,	1.63%
	DVL3,	2.39%
	SFRP1,	0.33%
	SFRP3 (FRZB),	0.54%
	GSK3β	0.93%
	TERT	25.62%
Hypoxia	HIF1A,	1.2%
	HIF2A (EPAS1)	0.72%
SHH	GLI2,	0.92%
	GLI3,	5.74%
	ISL1	0.65%
TGF-β	PDGFB,	1.41%
	NF-κB (RELA,NFKB),	0.65%
	NODAL,	0.82%
	SMAD2,	0.76%
	SMAD3,	0.82%
	SOX4,	0.82%
	SOX2,	4.12%
	LIF,	0.6%
	BMP7	0.6%
JAK-STAT	STAT3	1.05%
AKT	AKT1,	1.27%
	AKT2,	1.44%
	AKT3	1.27%

* Combined study (3060 samples) that encompassed glioblastoma (CPTAC, Cell 2021), glioblastoma multiform (TCGA, Firehose Legacy), diffuse glioma (GLASS Consortium), glioma (MSK, Clin Cancer Res 2019), brain lower grade glioma (TCGA, Firehose Legacy), glioblastoma (Columbia, Nat Med. 2019), diffuse glioma (MSK, Clin Cancer Res 2024).

**Table 2:** Clinical trials involving specific signaling inhibitors, from ClinicalTrials.gov database (https://clinicaltrials.gov/ accessed on 4 April 2024).

Signaling Component	Signaling Inhibitor	Phase/Identification Number/Status
EGFR	Epitinib	I/NCT03231501/unknown status
	BDTX-1535	I-II/NCT05256290/recruiting
WEE1	Adavosertib (AZD1775)	0-I/NCT02207010/completed
		I/NCT01849146/active, not recruiting
ALK, IGFR1, FAK	Ceritinib (LKD378)	0-I/NCT02605746/completed
TRK, ALK, ROS1	Entrectinib (Rxdx-101)	I-II/NCT02650401/active, not recruiting
CXCR4, MMP2 and MMP9	USL311	I-II/NCT02765165/terminated
		0-I/NCT03526822/recruiting
VEGFR, PDGFR, FGFR, Src	Ponatinib	II/NCT02478164/completed
	VXM01	I-II/NCT03750071/active, not recruiting
	NEO212	I-II/NCT06047379/recruiting
	Axitinib	II/NCT01562197/completed
	Bevacizumab and BKM120	I-II/NCT01349660/completed with results
		II/NCT01743950/recruiting
		I-II/NCT06011109/recruiting
	Erdafitinib	II/NCT05859334/recruiting
VEGFR, TIE-2, PDGFR, FGFR, KIT, RET, RAF	Regorafenib	II/NCT02926222/completed
	Anlotinib	I-II/NCT04004975/unknown status
		II/NCT04547855 unknown status
Src, VEGFR, c-MET	Dasatinib	I-II/NCT00892177/completed
		I/NCT01744652/completed
		I/NCT05432518/recruiting
	APL-101	II/NCT03175224/recruiting
BTK, Bruton’s tyrosine Kinase	Ibrutinib	I/NCT05106296/recruiting
STAT3	WP1066	I/NCT01904123/completed
		II/NCT05879250/not yet recruiting
AKT	Nelfinavir	I/NCT00694837/completed
mTOR	RMC-5552	NCT05557292/recruiting
GSK3beta	DSP-0390	I/NCT05023551/active not recruiting
